# Learning from the Physical Consequences of Our Actions Improves Motor Memory

**DOI:** 10.1523/ENEURO.0459-21.2022

**Published:** 2022-05-26

**Authors:** Amanda Bakkum, Daniel S. Marigold

**Affiliations:** 1 Department of Biomedical Physiology and Kinesiology, Simon Fraser University, Burnaby, British Columbia V5A 1S6, Canada; 2 Institute for Neuroscience and Neurotechnology, Simon Fraser University, Burnaby, British Columbia V5A 1S6, Canada

**Keywords:** balance, consolidation, generalization, locomotion, sensorimotor adaptation

## Abstract

Actions have consequences. Motor learning involves correcting actions that lead to movement errors and remembering these actions for future behavior. In most laboratory situations, movement errors have no physical consequences and simply indicate the progress of learning. Here, we asked how experiencing a physical consequence when making a movement error affects motor learning. Two groups of participants adapted to a new, prism-induced mapping between visual input and motor output while performing a precision walking task. Importantly, one group experienced an unexpected slip perturbation when making foot-placement errors during adaptation. Because of our innate drive for safety, and the fact that balance is fundamental to movement, we hypothesized that this experience would enhance motor memory. Learning generalized to different walking tasks to a greater extent in the group who experienced the adverse physical consequence. This group also showed faster relearning one week later despite exposure to a competing mapping during initial learning, evidence of greater memory consolidation. The group differences in generalization and consolidation occurred although they both experienced similar magnitude foot-placement errors and adapted at similar rates. Our results suggest the brain considers the potential physical consequences of movement error when learning and that balance-threatening consequences serve to enhance this process.

## Significance Statement

The ability to learn from past error is critical for safe and successful movement. In most laboratory situations, movement errors have no physical consequences and simply indicate the progress of learning. However, errors in daily life may cause harm to the individual: are such errors a stronger driver of learning compared with those that have no consequence? Using a walking adaptation paradigm, we show that experiencing a balance-threatening physical consequence when making errors improves motor memory. Our findings suggest that our motor systems may prioritize behaviors that promote personal safety and that we can exploit this notion to enhance motor learning. These findings also have intriguing implications for rehabilitation interventions.

## Introduction

Every action has a consequence. Different factors, such as reward and punishment, can serve to strengthen or reinforce the association between actions and their consequences and are therefore compelling modulators of behavior (Thorndike, 1933; Skinner, 1938; Abe et al., 2011; Galea et al., 2015). Motor learning involves correcting actions that lead to errors and remembering these actions for future performance. Sensory feedback plays an important role in this ability (Henriques and Cressman, 2012; Maeda et al., 2017a), although research also shows that punishing errors can accelerate motor learning whereas rewarding movement accuracy is beneficial for retaining motor memories (Wächter et al., 2009; Abe et al., 2011; Galea et al., 2015; Song and Smiley-Oyen, 2017; Quattrocchi et al., 2018; Hill et al., 2020). These experiments used monetary reward and punishment to reinforce learning, which does not reflect the movement consequences we experience in daily life. Rather, errors in everyday goal-directed movement often lead to physical consequences.

Some physical consequences of movement error are benign, while others have the potential to cause harm to the individual. An accidental misstep off a sidewalk, for example, can lead to an injurious fall. Thus, movement decisions are often made with personal safety in mind. Given that many daily movements are plagued by inherent stability challenges, a primary concern of the nervous system is to maintain balance. Even moderate perceived threats to postural stability elicit movement strategies that serve to safeguard balance (Adkin and Carpenter, 2018; Adkin et al., 2000; Brown et al., 2002; Manista and Ahmed, 2012). Such safety-driven movement strategies are also observed when walking across unstable terrain (Marigold and Patla, 2002). How might experiencing a balance-threatening physical consequence when making movement errors affect motor learning?

Evidence suggests that experiencing an unpleasant or dangerous physical consequence, or the threat of these types of consequences, can influence learning and memory. For example, rodents can quickly learn and subsequently remember the spatial location where foot shocks occur in an environment (Stuchlík et al., 2013; Willis et al., 2017). In addition, in humans, pairing an electric shock with images of neutral objects improves item recognition memory (Dunsmoor et al., 2015; Starita et al., 2019). Even the threat of being shocked enhances declarative memory (Murty et al., 2012). Because of our innate drive for safety, and the fact that balance is fundamental to movement, we hypothesized that experiencing a balance-threatening physical consequence when making a movement error would enhance motor memory.

To test this hypothesis, we had two groups of participants adapt to a new visuomotor mapping induced by prism lenses while performing a precision walking task that required them to step accurately to a target. Prism lenses cause errors in movement because they alter the normal relationship (or mapping) between visual input and motor output. Learning this mapping is thus essential for achieving movement accuracy. The groups differed in terms of the consequence experienced when making foot-placement errors to the target. Specifically, one group experienced a balance-threatening slip perturbation caused by stepping on a concealed slippery surface positioned adjacent to the target. In contrast to an artificial consequence, like monetary gains or losses, this slip perturbation is an adverse physical consequence that can occur in daily life.

We determined how the balance-threatening physical consequence affected (1) generalization of the learned visuomotor mapping across different visually guided walking tasks, and (2) consolidation of the learned mapping one week later. Generalization and consolidation are two hallmarks of motor memory (Poggio and Bizzi, 2004; Kitago and Krakauer, 2013; Krakauer et al., 2019). Here, we found that the group who experienced the adverse physical consequence better generalized learning to different walking tasks and showed greater consolidation. These results suggest our motor systems are tuned to remember motor behaviors that promote personal safety. These results may also help shape new neurorehabilitation strategies.

## Materials and Methods

### Participants

Twenty-four participants (mean age ± SD = 26.4 ± 5.0 years; 11 men, 13 women) with no known musculoskeletal, visual (six participants wore corrective lenses or glasses), or neurologic disease participated in this study. We randomly assigned these participants to one of two groups (*n* = 12 each; detailed below). We did not perform any a priori power analysis to determine sample size. Rather, we used sample sizes typical in the literature for this type of research (∼6–14 participants per group). The Office of Research Ethics at Simon Fraser University approved the study protocol, and all participants provided written informed consent before their participation.

### Experimental tasks and data collection

**Figure 1.**
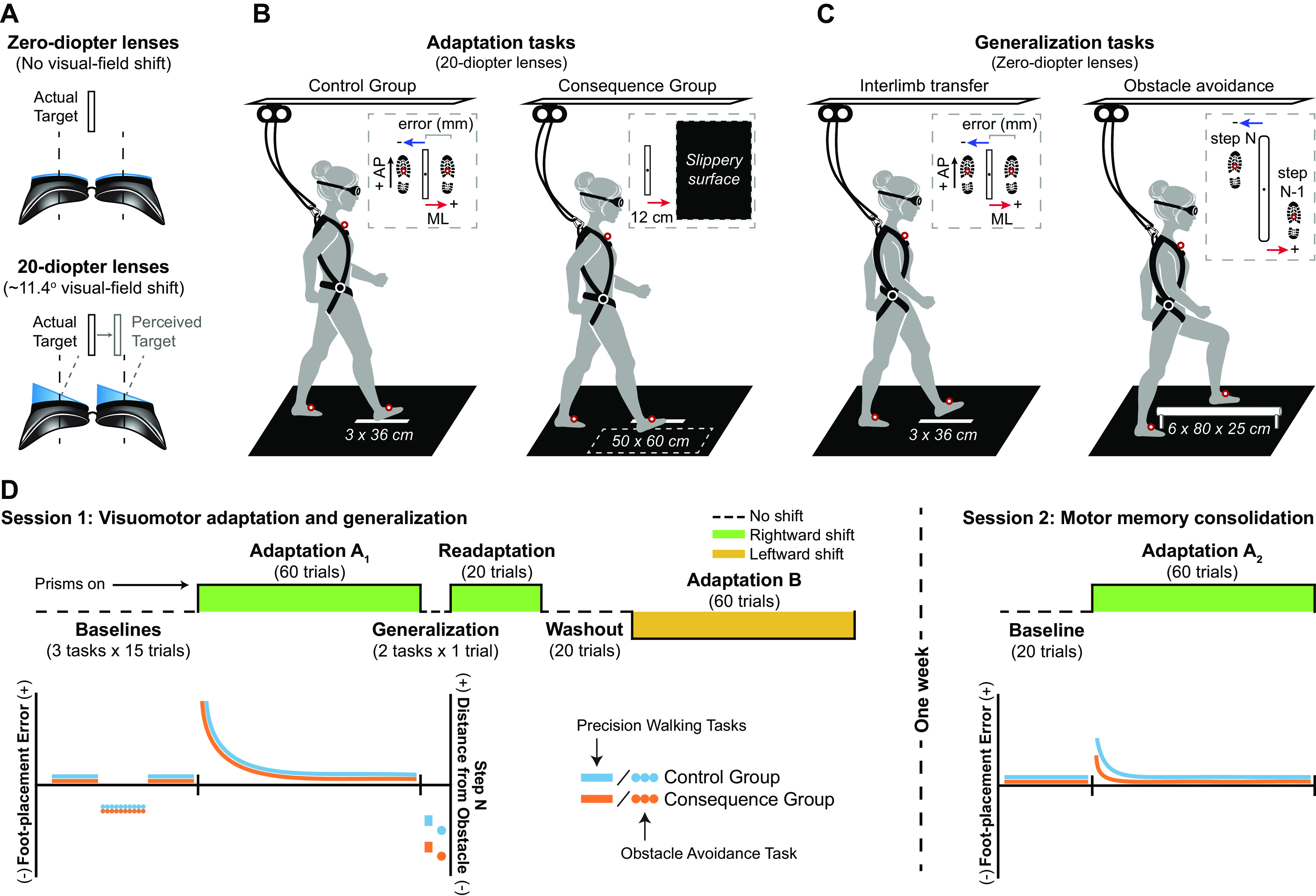
Experimental tasks and protocol. ***A***, A simulated view of the stepping target through the goggles coupled with zero-diopter (nonvisual-field-shifting) lenses and 20-diopter prism lenses that shift the perceived location of the target 11.4° to the right. ***B***, An illustration of the precision walking task without and with an adverse physical consequence, a slippery surface, present next to the stepping target. Inset, Left, A diagram showing positive (+) and negative (–) ML foot-placement error, defined as the distance between the position marker on the mid-foot and the center of the target. AP, anterior-posterior dimension in laboratory space. Inset, Right, Bird’s eye view of the location of the slippery surface relative to the target. ***C***, An illustration of the generalization tasks. Left, Interlimb transfer test. Note that the left foot is used to step to the target in this task. Inset, Identical to that shown in ***C***. Right, Obstacle avoidance task. Inset, A diagram showing positive (+) and negative (–) ML deviation from the obstacle, defined as the distance between the center of the obstacle and the position marker on the mid-foot of both the trailing foot (i.e., step N–1: right foot) and leading foot (i.e., step N: left foot). ***D***, An illustration of the experimental protocol across both testing sessions (top), as well as predicted foot-placement error (and distance from obstacle) profiles related to initial adaptation, generalization, and consolidation (bottom). Note that for the obstacle avoidance task, only predictions for step N are shown for simplicity. Generalization is evident from a negative (leftward) shift (i.e., opposite to the prism shift participants adapted to earlier) in error or distance. During the first testing session, all participants performed baseline, adaptation, generalization, readaptation, and washout phases. The baseline phase included the precision walking task present in the adaptation phase and the two generalization walking tasks. Depending on the phase, participants wore goggles paired with either zero-diopter or 20-diopter lenses. To assess consolidation, participants repeated baseline and adaptation phases one week later.

All participants adapted to a novel visuomotor mapping induced by prism lenses (Fig. 1*A*) while performing a precision walking task (Fig. 1*B*). For this task, participants stood at the beginning of the walkway (∼6 m in length) and waited for a go-cue to signal the start of each trial. Once cued, participants took a minimum of two steps before stepping with their right foot onto the medial-lateral (ML) center of a projected target (3 × 36 cm) without stopping. We instructed participants to be as accurate as possible in the ML dimension when stepping to the target. We used a long target to reduce the demand for accuracy in the anterior-posterior (AP) dimension and to prevent participants from using shuffle steps as they approached the target. We displayed the target in the center of the walking path using an LCD projector (Epson PowerLight 5535U; brightness of 5500 lumens). Participants performed the task under reduced light conditions (∼0.9 lux) to minimize the influence of environmental references and increase the visibility of the projected target.

We tested (1) generalization of the learned visuomotor mapping across different visually guided walking tasks, and (2) consolidation of the learned mapping one week later. If generalization is robust, it should be evident in a range of different walking tasks. We assessed generalization during an interlimb transfer test and obstacle-avoidance task performed without the prism lenses. Interlimb transfer tests are commonly used in reaching experiments (Wang, 2008). The ability to negotiate over or around obstacles (e.g., stairs, curbs, toys on the ground) is essential for safe mobility, and other prism adaptation studies have shown that people can generalize from a precision walking task to an obstacle avoidance task (Alexander et al., 2011, 2013).

During the interlimb transfer test, participants performed a single trial of the precision walking task using their left foot instead of their right foot to step to the target (Fig. 1*C*). Note that all participants stepped with the correct foot during the precision walking task. For the obstacle avoidance task, participants walked along the same 6-m-long path toward the right side of an obstacle (width = 6 cm; length = 80 cm; height = 25 cm) positioned in the center of the walkway. Once participants were beside the obstacle, they stepped laterally over the middle of it, first with their left leg (i.e., the leading leg), then their right leg (i.e., the trailing leg), before continuing to walk for several more steps (Fig. 1*C*). We instructed participants to avoid colliding with the obstacle.

We tracked body motion from infrared-emitting position markers placed on the participant’s chest (in line with the sternum) and bilaterally on the mid-foot (second to third metatarsal head) at 120 Hz during each task using an Optotrak Certus motion capture camera (Northern Digital). To mitigate adaptation between trials, we instructed participants to only have their eyes open when they performed the walking tasks. An experimenter guided the participants back to the beginning of the walkway between trials while their eyes were closed. To prevent participants from learning a specific stepping sequence and increase the demand for visual feedback, we randomized the AP starting location (between 1.5 and 2.5 m) for each trial. We also encouraged participants to perform each task at a quick and constant pace to minimize online corrections of leg trajectory during the step to the target. Participants walked with an average speed (±SD) of 1.56 ± 0.16 m/s, and we later verified the absence of these corrections by looking at the kinematic profiles of the mid-foot position markers. An experimenter demonstrated all tasks at the beginning of each testing session. Participants wore a safety harness at all times to prevent falling to the ground. No participant engaged the system during the experiment.

### Experimental protocol

We measured sensorimotor adaptation, generalization, and consolidation over two testing sessions, separated by one week. Figure 1*D* illustrates the experimental protocol and the predicted foot-placement error (and distance to obstacle) responses. Depending on the phase, participants wore goggles coupled with either zero-diopter (nonvisual-field-shifting) or 20-diopter prism lenses (Fig. 1*A*). The goggles block part of the peripheral visual field and participants had no option but to look through the lenses during each task. First, participants performed three baseline phases (15 trials each), one for each visually guided walking task, while wearing the zero-diopter (i.e., nonvisual-field-shifting) lenses. Participants performed the baseline phase for the adaptation task last, just before the adaptation phase. We counterbalanced the order of the two remaining baseline tasks for each participant and matched this order for the generalization tasks. Thereafter, participants performed the adaptation and generalization phases.

During the first adaptation phase (Adaptation A_1_), participants learned a new visuomotor mapping induced by the 20-diopter prism lenses (Fig. 1*A*) while performing 60 trials of the precision walking task using their right foot to step to the target. Participants adapted to the new visuomotor mapping with (consequence group; *n* = 12) or without (control group; *n* = 12) the possibility of experiencing an unexpected slip perturbation when making foot-placement errors. For the consequence group, we positioned a low-friction, polypropylene surface (50 × 60 cm) to the right of the target during prism exposure (Fig. 1*B*). We concealed this slippery surface using a solid black, low-friction fabric that covered the entire walking path. Exposure to the prism lenses induced a rightward deviation in foot placement to the target. This increased the likelihood of participants in the consequence group making contact with the slippery surface. On contact, the shear forces under the participants’ shoe at heel strike cause the low-friction fabric to slide over the slippery surface (kinetic coefficient of friction ≈ 0.09 μ_k_; for reference: ice ≈ 0.02 μ_k_) and require a reactive response to prevent falling. Participants only experienced the slip perturbation during stepping errors that were large enough that the foot made contact with the slippery surface. To prevent participants from being penalized during stepping errors within the normal range of late prism adaptation, we positioned the slippery surface 12 cm from the center of the target (Fig. 1*B*). We refer to late adaptation here as the period where performance plateaus indicating that the participant has likely adapted to the visuomotor shift. We based this margin for error on previous studies of prism adaptation during walking (Maeda et al., 2017a). A textured polyvinyl chloride bottom prevented the slippery surface from sliding along the walkway during foot contact.

Following adaptation to mapping A, participants performed the interlimb transfer test and the obstacle avoidance task (Fig. 1*C*,*D*) without the prism lenses to determine whether the learned mapping was applied to the nonadapted tasks. Participants then performed 20 readaptation trials while wearing the rightward-shifting 20-diopter lenses to mitigate any deadaptation that might have occurred during the generalization phase. To confirm whether the learned mapping was stored, participants performed 20 washout trials of the adaptation task with the zero-diopter lenses. Finally, ∼15 min after adaptation, participants performed 60 trials of the same adaptation task (i.e., precision walking with the right foot stepping on the target) while wearing 20-diopter lenses that shifted the visual field in the opposite (i.e., leftward) direction of mapping A, we refer to this as mapping B (Fig. 1*D*). Following the initial testing session, the participants returned to the lab one week later so we could probe motor memory consolidation. We define consolidation as memory stabilization of the learned prism-induced mapping such that it is resistant to retrograde interference by another (competing) mapping (Krakauer et al., 2005). Participants first performed 20 baseline trials of the adaptation task while wearing the zero-diopter lenses. Thereafter, all participants repeated the 60 adaptation trials with the 20-diopter prism lenses to assess consolidation of mapping A. There was no slippery surface present for either group during the second testing session.

### Data and statistical analyses

We analyzed kinematic data (filtered using a fourth-order, low-pass Butterworth algorithm with a cutoff frequency of 6 Hz) using MATLAB (The MathWorks) to calculate foot placement during the precision walking and obstacle avoidance tasks. We determined foot placement during each task as the moment of heel strike, derived using the vertical velocity of the mid-foot markers (O’Connor et al., 2007). For the precision walking task, we defined foot-placement error during the step to the target as the ML distance between the position of the mid-foot marker at heel strike and the center of the target. A positive value represents an error to the right of the target and a negative value represents an error to the left of the target (Fig. 1*B*). For the obstacle avoidance task, we calculated the ML distance between the obstacle and both the trailing foot (i.e., step N–1: right foot) and leading foot (i.e., step N: left foot) at heel strike using the mid-foot marker on each foot (Fig. 1*C*). For step N–1, increasing positive values represent a greater deviation of the right foot from the obstacle, whereas for step N, increasing negative values indicate greater deviation of the left foot from the obstacle.

During slip perturbations, we expected to see greater forward displacement and velocity of the right foot compared with baseline. Therefore, to test whether our hidden surface was effective at eliciting a slip, we calculated two measures for baseline and adaptation phase trials to quantify slip severity: slip distance and peak slip velocity. We calculated slip distance during the step to the target as the total AP displacement traveled by the right mid-foot marker between heel strike and slip end, the latter of which we defined as the moment AP velocity of the right mid-foot marker profile stabilized to zero. Note that AP displacement and velocity of the mid-foot marker has not stabilized to zero at heel strike; thus, we see a non-zero slip distance/velocity even for nonslip trials. We then calculated peak slip velocity as the maximum AP velocity of the right mid-foot marker within that same time interval (i.e., heel strike to slip end). We defined a slip perturbation trial, for each participant individually, as a slip distance or peak slip velocity greater than the mean plus 2 SDs of the last 10 baseline trials. To determine differences in slip severity, we compared slip distance and slip velocity during the baseline phase (mean of the last 10 trials) and the first adaptation trial between groups using separate two-way (Group × Phase) mixed-model ANOVAs, where we included participant as a random effect.

To determine how the adverse physical consequence associated with making an error affected adaptation, we compared foot-placement error during the baseline phase (mean of the last 10 trials), first adaptation trial, late adaptation (mean of the last 10 trials), and the first washout trial in the first testing session using a two-way (Group × Phase) mixed-model ANOVA (with participant as a random effect). When checking for the assumptions of an ANOVA, we found a potential outlier for the control group (studentized residual = 4.5). Excluding this data point did not change the results, suggesting it was noninfluential. Thus, we included this data point in the final statistical model.

To determine whether the learned visuomotor mapping generalized to the nonadapted limb during the precision walking task, we compared foot-placement error during the baseline phase when using the left foot to step to the target (mean of the last 10 trials) and the generalization trial with a two-way (Group × Phase) mixed-model ANOVA (with participant as a random effect). Foot-placement errors in the direction opposite to the learned prism shift (i.e., a negative value) indicate generalization during the precision walking task. To determine whether the learned visuomotor mapping generalized to the obstacle avoidance task, we compared foot-placement deviation from the obstacle during the baseline phase of this task (mean of the last 10 trials) and the generalization trial for both the trailing foot (i.e., step N–1: right foot) and leading foot (i.e., step N: left foot). Foot placement relative to the obstacle in the direction opposite to the learned prism shift (i.e., to the left) indicates generalization during the obstacle avoidance task. Thus, for step N–1 (right foot), a smaller value reflects less deviation of the foot from the obstacle (i.e., a leftward shift in foot placement; Fig. 1*C*) and indicates generalization of the learned mapping to the trailing foot. For step N (left foot), a greater negative value reflects greater deviation of the foot from the obstacle (i.e., a leftward shift in foot-placement; Fig. 1*C*) and indicates generalization of the learned mapping to the leading foot. We used separate two-way (Group × Phase) mixed-model ANOVAs (with participant as a random effect) to determine whether the learned mapping generalized to the obstacle avoidance task.

To determine the presence of consolidation, we quantified three measures: the first adaptation trial error, early adaptation error (i.e., mean of adaptation trials 2–8), and rate of adaptation. The first adaptation trial error represents the initial recall of the mapping. Early adaptation error captures the rapid reduction in foot-placement error early in the adaptation phase and does not assume that participants follow a specific pattern (i.e., it is a model-free measure; Malone et al., 2011; Roemmich and Bastian, 2015; Maeda et al., 2017b). A faster reduction in foot-placement error (i.e., faster relearning of the mapping, or savings) indicates consolidation of the learned mapping. Since model-based measures are also commonly used to quantify adaptation and savings (Morton and Bastian, 2004; Criscimagna-Hemminger and Shadmehr, 2008), we also calculated the rate of adaptation. This involved fitting an exponential model to the foot-placement error data during the 60 adaptation trials associated with mapping A for each testing session using the following equation:

(1) $$y=a-b\times e^{-x/c},$$

where, *a* is the residual error after asymptote (i.e., steady state), *b* is the magnitude of the adaptation required to reach *a* from the first trial, *c* is the decay constant, and *x* is the trial number. We defined the rate of adaptation as the number of trials taken to reach ∼63.2% of adaptation (Martin et al., 1996). We used separate two-way (Group × Session) mixed-model ANOVAs (with participant as a random effect) to determine differences in first adaptation trial error, early adaptation error, and adaptation rates between groups.

We used JMP 15 software (SAS Institute Inc.) with an α level of 0.05 for all statistical analyses. For ANOVAs, we used Tukey’s *post hoc* tests, where appropriate, when we found significant main effects or interactions. We report effect sizes as $\eta_{p}^{2}$.

### Data availability

Data used to make figures is available on Open Science Framework (https://osf.io/9tc5s/).

## Results

### Contact with the slippery surface elicited a slip perturbation

**Figure 2.**
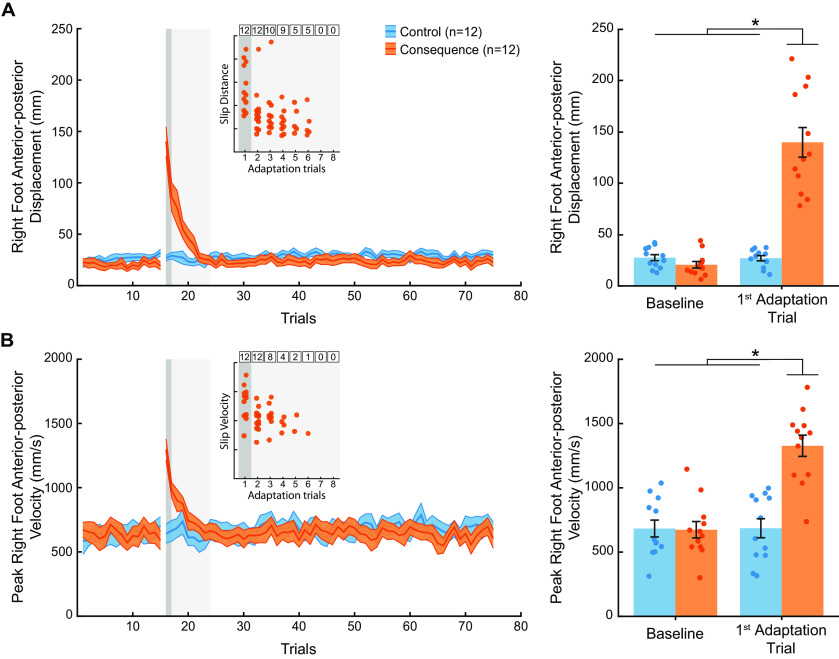
Slip measures. ***A***, Left, Group mean ± SE slip distance across all trials for baseline and adaptation phases for the control (blue) and consequence (orange) groups. Right, Group mean ± SE slip distance for the baseline phase (mean of the last 10 trials) and the first adaptation trial for the control (blue) and consequence (orange) groups. ***B***, Left, Group mean ± SE peak slip velocity across all trials for baseline and adaptation phases for the control (blue) and consequence (orange) groups. Right, Group mean ± SE peak slip velocity for the baseline phase (mean of the last 10 trials) and the first adaptation trial for the control (blue) and consequence (orange) groups. Individual participant values are superimposed. * Indicates that values are significantly different from each other based on *post hoc* tests (*p* < 0.05). See Extended Data Figure 2-1 for more detailed *post hoc* test results. Insets, Individual data points showing significant evidence of a slip perturbation, defined as a slip distance or slip velocity of greater than the mean plus 2 SDs of the last 10 baseline trials. The dark gray shaded box represents the first adaptation trial, and the light gray shaded box represents early adaptation trials. The numbered black boxes represent how many participants slipped during each trial. Every participant in the consequence group slipped during the first two adaptation trials. No participants slipped after the sixth adaptation trial.

To confirm that participants in the consequence group experienced a slip perturbation when missing the target, we calculated measures of slip distance and slip velocity during the baseline and adaptation phases for the consequence and control groups. Figure 2 illustrates group mean slip distance and peak slip velocity. We found that every participant in the consequence group (*n* = 12) experienced a slip during the first adaptation trial, which we define, for each participant individually, as a slip distance or peak slip velocity greater than the mean plus 2 SDs of the last 10 trials of the baseline phase. During the first adaptation trial, the consequence group demonstrated a significantly greater slip distance compared with baseline and the control group (Fig. 2*A*; Extended Data Fig. 2-1; mixed-model ANOVA, Group × Phase interaction: *F*_(1,22)_ = 85.49, *p* = 4.927e-9, $\eta_{p}^{2}$= 0.80), reflecting greater forward displacement of the right foot after contact with the slippery surface. Similarly, we found that peak slip velocity was significantly greater for the consequence group during the first adaptation trial compared with their baseline trials and the control group (Fig. 2*B*; Extended Data Fig. 2-1; mixed-model ANOVA, Group × Phase interaction: *F*_(1,22)_ = 34.85, *p* = 6.103e-6, $\eta_{p}^{2}$= 0.61). Additionally, all participants in the consequence group slipped during the second adaptation trial. Thereafter, the number of slips declined, and no participants slipped after the sixth adaptation trial. We found that the number of slips per trial differed slightly depending on the slip measure (i.e., slip distance or slip velocity), although this is likely because of the variability of the peak slip velocity measure. Overall, contact with the slippery surface during the precision walking task successfully elicited an adverse physical consequence, that is, a slip perturbation.

10.1523/ENEURO.0459-21.2022.f2-1 Extended Data Figure 2-1 Least square means Tukey’s HSD tests of pairwise comparisons related to Figure 2. A, Pairwise comparisons for slip distance following a statistically significant Group × Phase interaction. B, Pairwise comparisons for slip velocity following a statistically significant Group × Phase interaction. Values are shown as mean differences with 95% confidence intervals and p values. Download Figure 2-1, EPS file.

### The presence of the adverse physical consequence did not disrupt initial visuomotor adaptation

**Figure 3.**
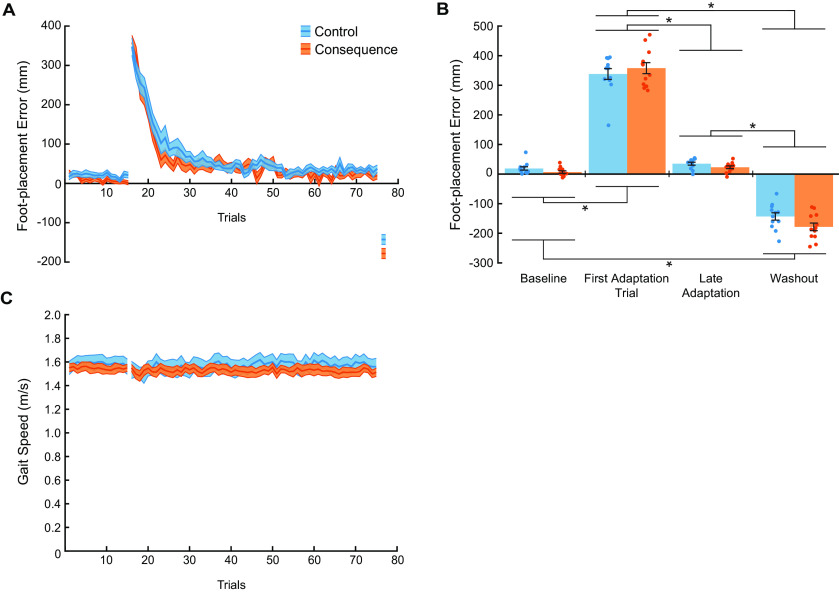
Visuomotor adaptation during session 1. ***A***, Group mean ± SE ML foot-placement error across all trials for baseline and adaptation phases and the first washout trial during the first testing session for the control (blue) and consequence (orange) groups. ***B***, Group mean ± SE foot-placement error for the baseline phase (mean of the last 10 trials), first adaptation trial, late adaptation (mean of the last 10 trials), and first washout trial during the first testing session for the control (blue) and consequence (orange) groups. Individual participant values are superimposed (*n* = 12 per group). A positive value represents errors in the direction of the prism shift (i.e., to the right of the target) and negative values represent errors in the opposite direction to the prism shift. ***C***, Group mean ± SE gait speed during the precision walking task. * Indicates that values are significantly different from each other based on *post hoc* tests (*p* < 0.05). See Extended Data Figure 3-1 for more detailed *post hoc* test results.

Upon initial exposure to the prisms, all participants demonstrated a large, rightward deviation in foot placement relative to the target during the precision walking task. As participants adapted to the new, prism-induced visuomotor mapping, foot-placement error gradually returned to near-baseline levels of performance. Upon removal of the prism lenses, participants demonstrated a large, leftward deviation in foot-placement error (i.e., a negative aftereffect). These results are illustrated in Figure 3*A*.

10.1523/ENEURO.0459-21.2022.f3-1 Extended Data Figure 3-1 Least square means Tukey’s HSD tests of pairwise comparisons related to Figure 3. Pairwise comparisons for foot-placement error following a statistically significant main effect of Phase. Values are shown as mean differences with 95% confidence intervals and p values. Download Figure 3-1, EPS file.

To determine the effects of the adverse physical consequence on visuomotor adaptation, we compared foot-placement error across the baseline phase (mean of last 10 trials), first adaptation trial, late adaptation (mean of last 10 trials), and the first washout trial across groups during the first testing session. We found that foot-placement error differed depending on the phase (Fig. 3*B*; mixed-model ANOVA, Phase main effect: *F*_(3,88)_ = 657.4, *p* = 4.11e-60, $\eta_{p}^{2}$= 0.96). *Post hoc* tests indicated significantly greater foot-placement error during the first adaptation trial compared with the other phases (see also Extended Data Fig. 3-1). Furthermore, foot-placement error during the first washout trial differed significantly from the other testing phases. We did not detect significant differences between the control and consequence groups across the testing phases. Overall, the adverse physical consequence experienced when making foot-placement errors did not affect the ability to adapt to the new, prism-induced visuomotor mapping.

It is of interest to note that gait speed remained consistent across the baseline and adaptation phase trials for both groups. This is shown in Figure 3*C*. Also evident in this figure is the similar gait speeds between groups. To confirm this observation, we ran a two-way, mixed-model ANOVA on the mean gait speed in each phase between the two groups. We found no significant difference in the gait speeds between groups or across the baseline and adaptation phases (Group main effect: *F*_(1,22)_ = 1.0, *p* = 3.221e-1, $\eta_{p}^{2}$ = 0.04, Phase main effect: *F*_(1,22)_ = 0.95, *p* = 3.405e-1, $\eta_{p}^{2}$ = 0.04, Group × Phase interaction: *F*_(1,22)_ = 0.36, *p* = 5.565e-1, $\eta_{p}^{2}$ = 0.02).

### Learning generalized to a greater extent in the group who experienced the adverse physical consequence

To determine whether the learned mapping generalized to nonadapted tasks, we had participants perform an interlimb transfer test and an obstacle-avoidance task following the initial adaptation to mapping A (Fig. 1*C*,*D*). During the interlimb transfer test, participants performed a single trial of the precision walking task using their left foot instead of their right foot to step to the target. For the obstacle avoidance task, participants walked toward the right side of an obstacle positioned in the center of the walkway and subsequently stepped laterally over it, first with their left leg (i.e., the leading leg), then their right leg (i.e., the trailing leg), before walking for several more steps (Fig. 1*C*, right).

**Figure 4.**
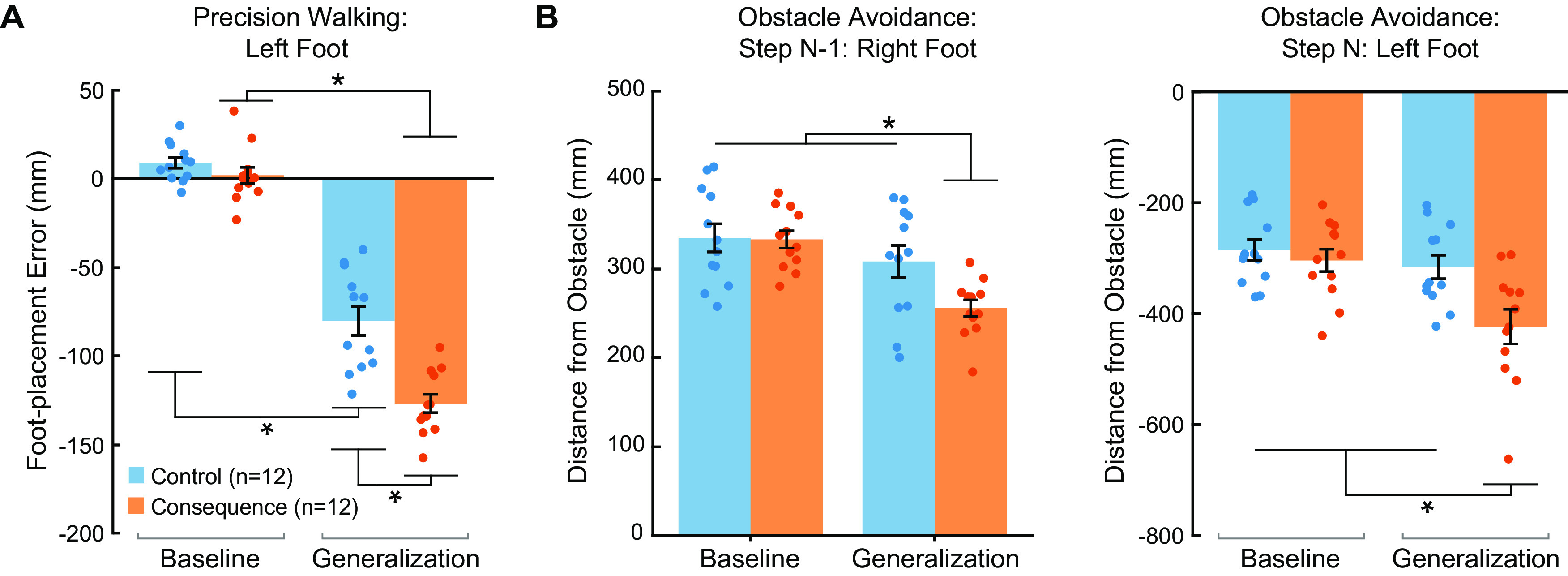
Generalization. ***A***, Group mean ± SE foot-placement error for the baseline (mean of the last 10 trials) and generalization phases during the precision walking task for the control (blue) and consequence (orange) groups. Foot-placement errors in the direction opposite to the prism shift (i.e., a negative value) indicate generalization. ***B***, Group mean ± SE foot-placement error for the baseline (mean of the last 10 trials) and generalization phases during the obstacle avoidance task for both the trailing foot (i.e., step N–1: right foot) and leading foot (i.e., step N: left foot) for the control (blue) and consequence (orange) groups. A smaller value indicates generalization for step N–1 (right foot), whereas a greater negative value reflects generalization for step N (left foot). Individual participant values are superimposed (*n* = 12 per group). * Indicates that values are significantly different from each other based on *post hoc* tests (*p* < 0.05). See Extended Data Figure 4-1 for more detailed *post hoc* test results.

To determine whether the learned visuomotor mapping generalized to the left leg/foot, we compared the mean foot-placement error during the last 10 baseline trials (when using the left foot to step to the target) to the foot-placement error during the generalization trial. We found that both the control and consequence groups generalized the learned mapping to the nonadapted left foot (mixed-model ANOVA, Group × Phase interaction: *F*_(1,22)_ = 12.70, *p* = 0.002, $\eta_{p}^{2}$ = 0.37), reflected by foot-placement errors in the direction opposite to the learned prism shift (i.e., a negative value; Fig. 4*A*). However, the foot-placement error during the generalization trial differed significantly between groups, such that the consequence group demonstrated greater leftward deviation in foot placement from the target (see also Extended Data Fig. 4-1), indicating greater generalization to the left leg/foot during precision walking.

10.1523/ENEURO.0459-21.2022.f4-1 Extended Data Figure 4-1 Least square means Tukey’s HSD tests of pairwise comparisons related to Figure 4. A, Pairwise comparisons for foot-placement error in the interlimb transfer test following a statistically significant Group × Phase interaction. B, Pairwise comparisons for foot placement of step N–1 (right foot) relative to the obstacle following a statistically significant Group × Phase interaction. C, Pairwise comparisons for foot placement of step N (left foot) relative to the obstacle following a statistically significant Group × Phase interaction. Values are shown as mean differences with 95% confidence intervals and p values. Download Figure 4-1, EPS file.

For the obstacle avoidance task, we compared foot-placement deviation from the obstacle during the baseline phase (mean of last 10 trials) and the generalization trial for both the trailing foot (i.e., step N–1: right foot) and leading foot (i.e., step N: left foot). For step N–1 (right foot), a smaller value reflects less deviation of the foot from the obstacle and indicates generalization of the learned mapping to the trailing right foot. We found that the consequence group demonstrated a smaller deviation of the right foot from the obstacle (i.e., a leftward shift in foot placement) during step N–1 compared with the control group (Fig. 4*B*; Extended Data Fig. 4-1; mixed-model ANOVA, Group × Phase interaction: *F*_(1,22)_ = 10.98, *p* = 0.003, $\eta_{p}^{2}$ = 0.33). For step N (left foot), a greater negative value reflects greater deviation of the foot from the obstacle and indicates generalization of the learned mapping to the leading left foot. We found that the consequence group demonstrated greater deviation of the leading left foot from the obstacle (Fig. 4*B*; Extended Data Fig. 4-1; mixed-model ANOVA, Group × Phase interaction: *F*_(1,22)_ = 16.18, *p* = 0.0006, $\eta_{p}^{2}$ = 0.42). Taken together, experiencing an adverse physical consequence when making movement errors increases the degree of generalization across different visually guided walking tasks.

### Greater motor memory consolidation occurred in the group who experienced the adverse physical consequence

We also probed consolidation, defined here as memory stabilization of the learned prism-induced mapping such that it is resistant to retrograde interference by another (competing) mapping (Krakauer et al., 2005). Thus, at the end of the first testing session, participants performed 60 trials of the same adaptation task (i.e., precision walking with the right foot stepping on the target) while wearing the 20-diopter lenses that shifted the visual field in the opposite (i.e., leftward) direction of mapping A (which we refer to as mapping B; Fig. 1*D*). Following the initial testing session, the participants returned to the lab one week later to probe motor memory consolidation. Participants first performed 20 baseline trials of the adaptation task while wearing the zero-diopter lenses. Thereafter, all participants repeated the 60 adaptation trials with the 20-diopter (rightward-shifting) prism lenses to assess consolidation of mapping A. There was no slippery surface (and hence no adverse physical consequence for making a movement error) during the second testing session.

**Figure 5.**
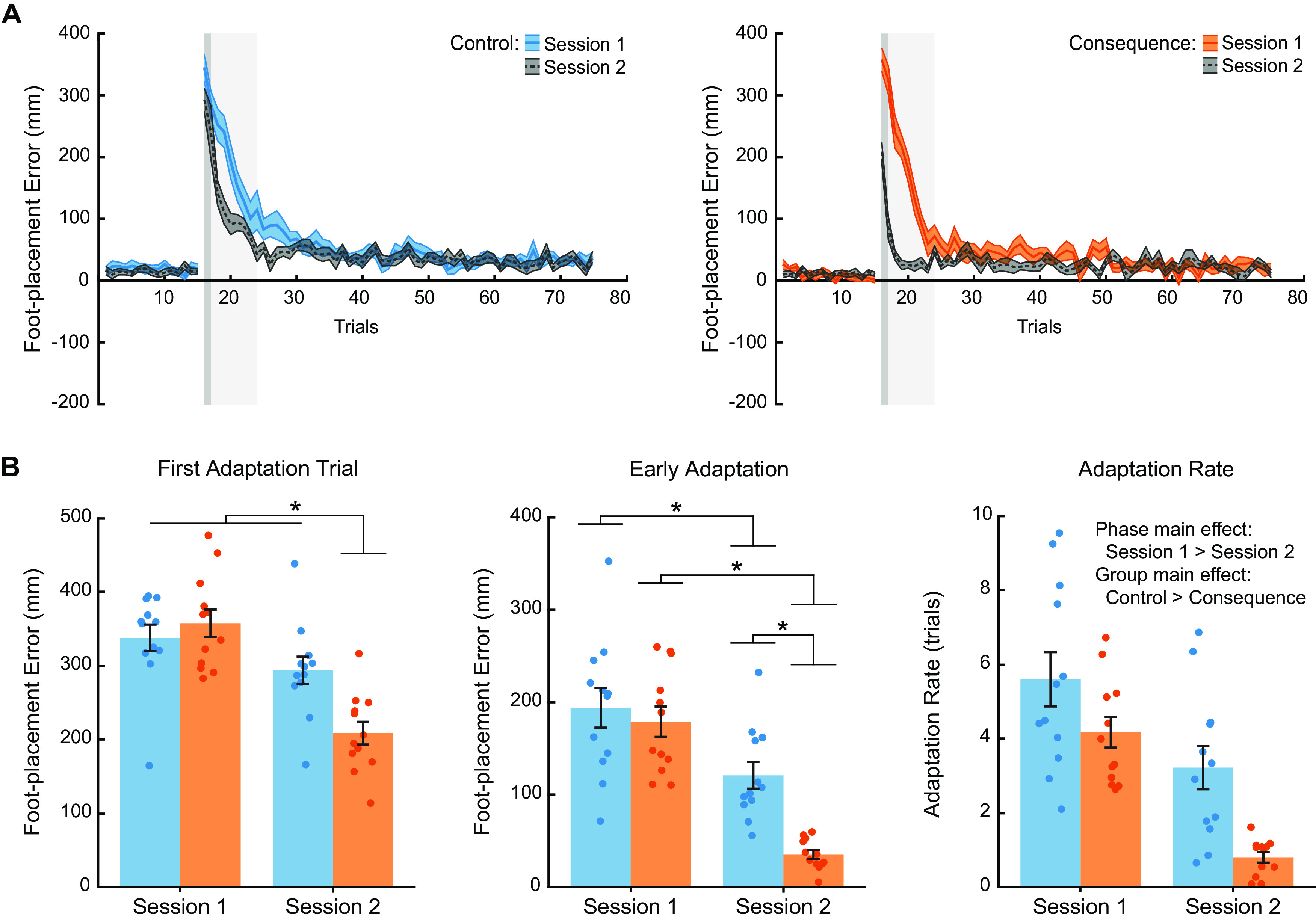
Motor memory consolidation. ***A***, Group mean ± SE foot-placement error for all trials in the baseline and adaptation phases across testing sessions for the control (blue) and consequence (orange) groups (*n* = 12 per group for both sessions). ***B***, Group mean ± SE for the first adaptation trial error (dark gray shaded box in panel a), early adaptation error (light gray shaded box in panel ***A***), and rate of adaptation across testing sessions for the control (blue) and consequence (orange) groups. One week separated testing sessions. Individual participant values are superimposed (*n* = 12 per group). * Indicates that values are significantly different from each other based on *post hoc* tests (*p* < 0.05). See also Extended Data Figure 5-1 for more detailed *post hoc* test results.

To determine the presence of consolidation, we compared three measures across testing sessions: the first adaptation trial error (representing the initial recall of the mapping), early adaptation error (i.e., mean of adaptation trials 2–8), and rate of adaptation. We used a single exponential for our adaptation rate measure. The *R*^2^ values of the individual fits in session 1 for the control group were 0.77 ± 0.09 (range: 0.60–0.90) and the consequence group were 0.79 ± 0.10 (range: 0.62–0.91). The *R*^2^ values of the individual fits in session 2 for the control group were 0.68 ± 0.16 (range: 0.38–0.89) and the consequence group were 0.53 ± 0.12 (range: 0.37–0.76). Figure 5*A* illustrates group mean foot-placement error during the baseline and adaptation phases during both testing sessions for the control and consequence groups. We found that the consequence group demonstrated greater error reduction in the first adaptation trial compared with the control group (mixed-model ANOVA, Group × Session interaction: *F*_(1,22)_ = 18.18, *p* = 0.0003, $\eta_{p}^{2}$ = 0.45), reflecting greater recall of the learned mapping one week later (Fig. 5*B*; Extended Data Fig. 5-1).

10.1523/ENEURO.0459-21.2022.f5-1 Extended Data Figure 5-1 Least square means Tukey’s HSD tests of pairwise comparisons and least square means plots related to Figure 5. A, Pairwise comparisons for first adaptation trial foot-placement error following a statistically significant Group × Session interaction. B, Pairwise comparisons for early adaptation foot-placement error following a statistically significant Group × Session interaction. C, Least square means plots for adaptation rate following statistically significant main effects. Values are shown as mean differences with 95% confidence intervals and p values for A, B. Values are shown as least square means with 95% confidence intervals and p values for C. S1 = session 1; S2 = session 2. Download Figure 5-1, EPS file.

A faster reduction in foot-placement error (i.e., savings) indicates that the learned mapping was consolidated. To quantify savings of the learned mapping, we compared foot-placement error during early adaptation (i.e., mean of adaptation trials 2–8) and the rate of adaptation across testing sessions. We found that both the control and consequence groups showed a significant reduction in foot-placement error during early adaptation (Fig. 5*B*; Extended Data Fig. 5-1; mixed-model ANOVA, Group × Session interaction: *F*_(1,22)_ = 7.98, *p* = 0.010, $\eta_{p}^{2}$ = 0.27). However, the consequence group demonstrated significantly greater error reduction during the second testing session compared with the control group, reflecting greater savings one week later. We also found that both the control and consequence groups demonstrated a faster rate of relearning during the second testing session (Fig. 5*B*; Extended Data Fig. 5-1; mixed-model ANOVA, Session main effect: *F*_(1,44)_ = 30.99, *p* = 1.454e-6, $\eta_{p}^{2}$ = 0.41). In addition, we found that the consequence group demonstrated a faster rate of adaptation across both testing sessions (mixed-model ANOVA, Group main effect: *F*_(1,44)_ = 13.86, *p* = 0.0006, $\eta_{p}^{2}$ = 0.24), although we did not detect a significant Group × Session interaction for this measure (mixed-model ANOVA, *F*_(1,44)_ = 0.92, *p* = 0.344, $\eta_{p}^{2}$ = 0.02). Taken together, experiencing an adverse physical consequence when making movement errors increases initial recall and savings of a learned mapping one week later, reflective of greater motor memory consolidation.

**Figure 6.**
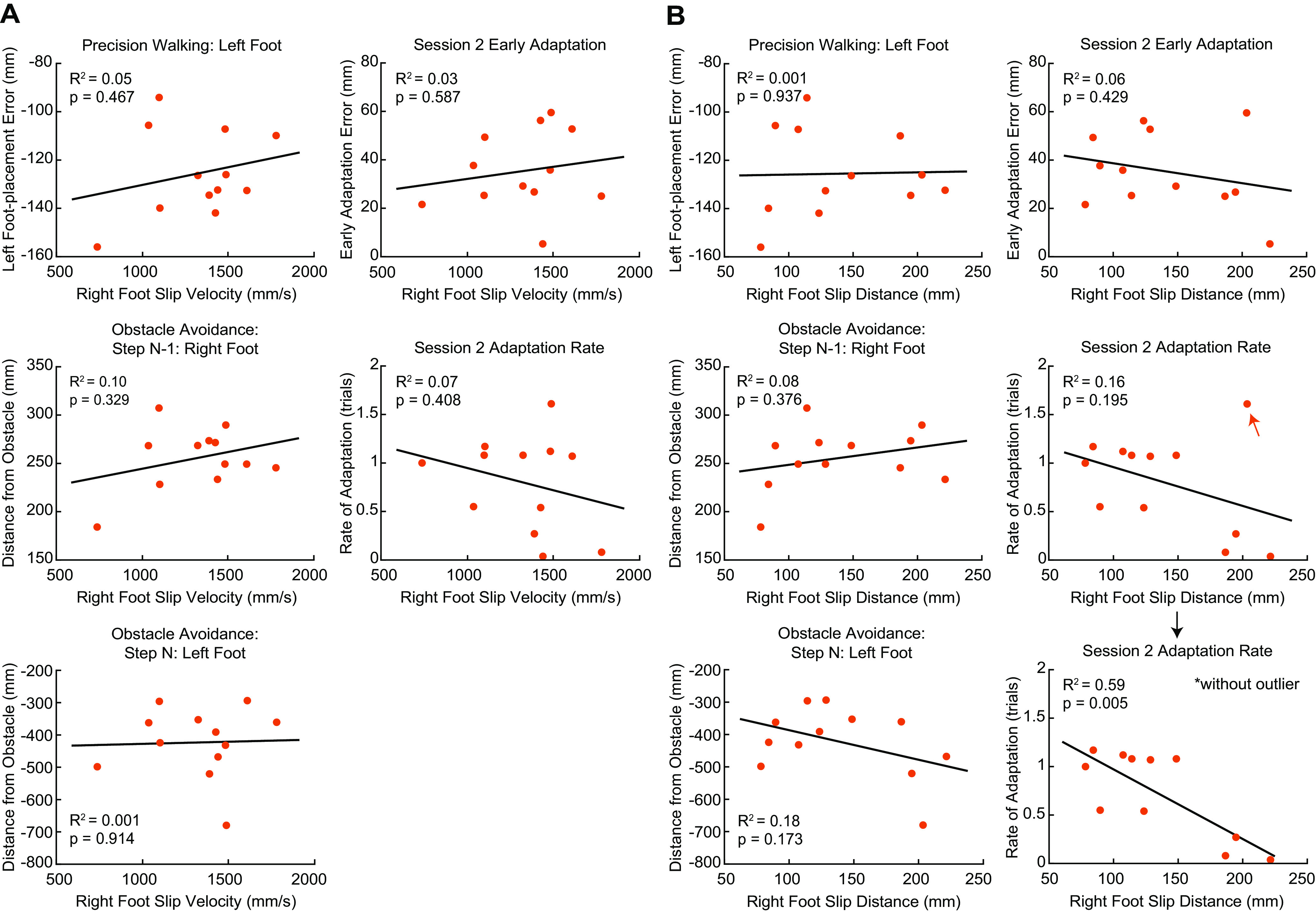
Relationship between slip severity and measures of generalization and consolidation. ***A***, Scatter plots of the relationship between slip velocity and measures of generalization (left column) and consolidation (right column). ***B***, Scatter plots of the relationship between slip distance and measures of generalization (left column) and consolidation (right column). Solid lines indicate the linear fits obtained from the regression analyses. See also Extended Data Figure 6-1.

As an exploratory analysis, we performed separate linear regression analyses to determine whether the severity of the first slip (i.e., slip velocity and slip distance during the first adaptation trial in session 1) predicted our measures of generalization and consolidation in the consequence group (Fig. 6; Extended Data Fig. 6-1). We did not detect a significant relationship between slip velocity and generalization for the interlimb transfer or the obstacle avoidance tasks (Fig. 6*A*, left panel). Likewise, we did not detect a significant relationship between slip velocity and the session 2 early adaptation error and adaptation rate (Fig. 6*A*, right panel). Similarly, we did not detect any significant relationships between slip distance and our measures for generalization (Fig. 6*B*, left panel) and consolidation (Fig. 6*B*, right panel). However, we noted a potential influential outlier (Cook’s D = 0.96; see arrow next to data point) when comparing slip distance with adaptation rate. Removing this outlier resulted in a significant relationship (*R*^2^ = 0.59, *p* = 0.005), which showed that greater slip distance during the first slip associates with faster adaptation in session 2. Taken together, however, we did not find strong evidence to suggest that slip severity predicts our results.

10.1523/ENEURO.0459-21.2022.f6-1 Extended Data Figure 6-1 Relationship between the number of slips during the adaptation phase and measures of generalization and consolidation. A, Scatter plots of the relationship between slip count and measures of generalization. B, Scatter plots of the relationship between slip count and measures of consolidation. Download Figure 6-1, EPS file.

## Discussion

Learning from the consequences of our actions is imperative for safe and successful motor performance. To determine which behaviors to maintain, people presumably learn to dissociate actions that give rise to desirable outcomes from those that do not. Here, we tested the hypothesis that experiencing a balance-threatening physical consequence when making a movement error serves to enhance motor memory. Previous research on reaching adaptation has reported limited generalization (Ghahramani et al., 1996; Krakauer et al., 2000; Wang, 2008) and mixed evidence for motor memory consolidation (Brashers-Krug et al., 1996; Caithness et al., 2004; Krakauer et al., 2005), although research on walking has found stronger evidence of both (Malone et al., 2011; Maeda et al., 2017b, 2018; Bakkum et al., 2020, 2021). In this study, we found that participants who experienced an adverse physical consequence when making foot-placement errors during adaptation demonstrated increased interlimb transfer on a precision walking task and generalization to an obstacle-avoidance task. Furthermore, this group showed increased recall and savings of the learned visuomotor mapping one week later despite exposure to a competing mapping during initial learning, evidence of greater motor memory consolidation. The differences in generalization and consolidation between groups occurred although they both experienced similar magnitude foot-placement errors and adapted at similar rates. Our results suggest that the brain considers the potential physical consequences of movement error when learning and that balance-threatening consequences serve to enhance this process.

Reward and punishment are known to reinforce motor learning. If one considers our physical consequence as punishment, then our results contrast with previous work that shows punishment accelerates adaptation rate but has little effect on later retention (Galea et al., 2015; Song and Smiley-Oyen, 2017; Song et al., 2020). On the other hand, avoiding the adverse physical consequence may serve as a reward. Specifically, as participants adapt to the prisms and become more accurate stepping to the target, they decrease the likelihood of contacting the slippery surface and suffering a potential loss of balance. Thus, foot placement on the target, or foot placement with minimal error such that the foot does not hit the slippery surface, may act as reward-like feedback. Interestingly, avoiding an aversive outcome causes activation in a brain region, medial orbitofrontal cortex, also implicated in encoding reward (Kim et al., 2006). In this case, our results are compatible with previous work that shows reward enhances retention but does not affect adaptation rate (Galea et al., 2015; Song and Smiley-Oyen, 2017), although it is important to note that reward can accelerate learning depending on the reward structure (Nikooyan and Ahmed, 2015). However, it may not be appropriate to associate our physical consequence with monetary reward or punishment per se. Rather, the slip serves as a functionally meaningful consequence of movement error, thus making it a more ecological manipulation.

Perhaps surprisingly, we found similar adaptation rates between groups in session 1, although the one group experienced the adverse physical consequence. However, there is minimal room for differences given that rates are already quite fast in this paradigm (i.e., less than six trials on average). Thus, the rapid nature of adaptation may have masked any potential group differences. In addition, the slip exposure does not provide information on how to adapt to the new prism-induced visuomotor mapping, which may further explain the lack of differences. Alternatively, our results suggest that the adverse physical consequence may simply influence the strength of the learned mapping. Specifically, since the control of balance is fundamental to movement, the brain may assign greater importance (or value) to maintaining the learned visuomotor mapping because it ensures the slip is avoided. Thus, just like expected value increases with the probability of reward, value may increase with the probability of maintaining balance.

**Figure 7.**
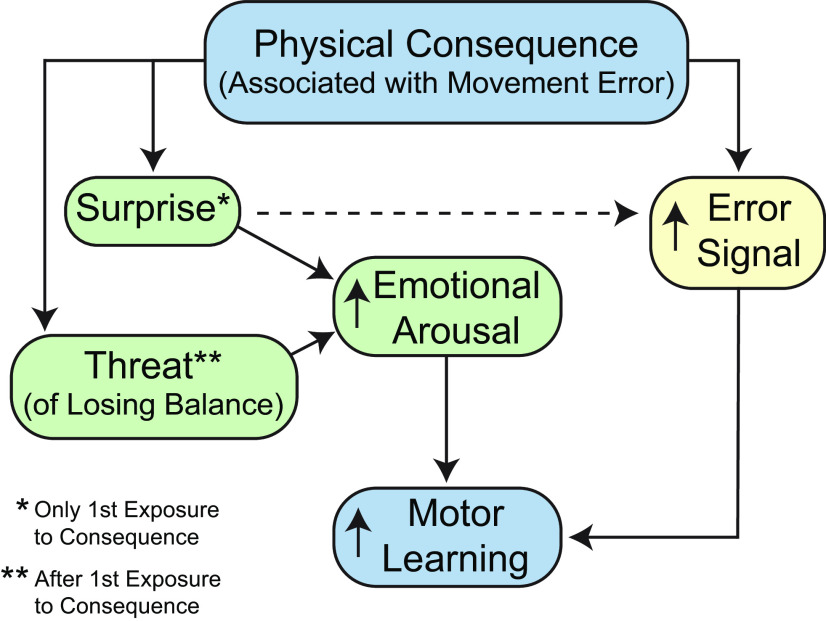
Summary of the possible mechanisms for the improvement in motor learning observed for the consequence group. The slip perturbation experienced when making a foot-placement error because of the novel visuomotor mapping may have served as a surprise (for the first exposure during adaptation) and increased the threat of losing balance (in subsequent walking trials). These factors may have increased emotional arousal, which led to a strengthening of synaptic connections in relevant sensorimotor areas where memory of the learned mapping was marked for consolidation. The slip perturbation associated with making foot-placement errors (possibly because of the surprising nature of it) may have also increased the error signal itself, leading to greater generalization and consolidation.

Our physical consequence threatened balance, and the unexpected nature of at least the first slip experience likely surprised participants. This latter idea may serve to increase the error signal itself. Both threat and surprise can increase emotional arousal. We propose that experiencing the adverse physical consequence when making foot-placement errors may have enhanced motor memory through increased recruitment of brain regions engaged in processing emotional arousal. Research in humans and other animals provides compelling evidence for the role of the amygdala in forming and maintaining lasting memories associated with emotional arousal (McGaugh, 2004). For example, in humans, the threat of being shocked enhances declarative memory through activation of the amygdala (Murty et al., 2012). Furthermore, lesions to the amygdala attenuate the advantageous effects of emotional arousal on memory (McGaugh et al., 1996). The locus coeruleus (LC), which is heavily connected to the amygdala, is also activated in response to emotionally arousing stimuli, including threat, and can facilitate memory encoding (Tully and Bolshakov, 2010; Clewett et al., 2018). In addition, the anterior cingulate cortex (ACC) is active in response to error detection or surprise (Hayden et al., 2011) and interestingly, shows greater electroencephalography-based theta band spectral power following a loss of balance during walking (Sipp et al., 2013). The amygdala, LC, and ACC connect directly or indirectly to the motor cortex, cerebellum, and basal ganglia (Tully and Bolshakov, 2010; Farley et al., 2016; Grèzes et al., 2014; Rolls, 2019; Schönfeld and Wojtecki, 2019), which are each implicated in motor memory consolidation (Debas et al., 2010; Landi et al., 2011; Leow et al., 2012). Thus, the emotionally charged experience of slipping may have increased the activation of one or more of these regions during adaptation and led to strengthening of synaptic connections in relevant sensorimotor areas where memory of the learned mapping was marked for consolidation. This idea resembles the emotional tagging hypothesis (Richter-Levin and Akirav, 2003; McReynolds and McIntyre, 2012), which attempts to explain how and why emotionally arousing events are better remembered. Figure 7 summarizes the possible mechanisms for the results of the consequence group.

The effects of the adverse physical consequence associated with movement error may derive from an implicit and/or explicit learning process. There is strong evidence that implicit, internal-model-based learning occurs in walking adaptation paradigms (Maeda et al., 2017a). However, recent work highlights the contribution of explicit strategies to reinforcement-based visuomotor adaptation (Codol et al., 2018; Holland et al., 2018; Song et al., 2020). Additionally, there is research that suggests that generalization is maximized around the intended location of an explicitly accessible motor plan (Day et al., 2016; McDougle et al., 2017) and that savings can be achieved explicitly through the recall of a deliberate aiming strategy (Haith et al., 2015; Morehead et al., 2015). Given the nature of our physical consequence, it may draw greater attention to the error and, thus, serve to increase the reliance on using an explicit aiming strategy to regain movement accuracy. For instance, it is well established that postural threat increases the reliance on conscious control (Huffman et al., 2009; Ellmers and Young, 2019; Ellmers et al., 2020). Additionally, while the consequence group does not demonstrate a significantly faster rate of adaptation during session 1, the adaptation profiles and rate data appear qualitatively different compared with the control group (see Fig. 5). This may indicate that participants in the consequence group are engaging more explicit strategies to help rapidly reduce movement errors and compensate for the visual perturbation. On the other hand, the consequence group demonstrates greater generalization for the precision walking task, reflected by increased foot-placement error (i.e., a negative aftereffect). This may indicate a more implicit learning process, as one would expect explicit strategies to override this increased error. Taken together, we suggest that it is likely a combination of both implicit-based and explicit-based learning, but the distinction between these processes is beyond the scope of this study.

Overall, our findings show that experiencing an adverse physical consequence when making errors, which threatens stability, enhances sensorimotor learning. Specifically, the consequence group better generalized learning to different walking tasks and showed greater consolidation one week later. This may suggest our motor systems are tuned to remember behaviors that promote personal safety and provide an important survival advantage (Nairne et al., 2007). Our work highlights a new factor that affects sensorimotor learning and provides an interesting avenue for future research. Our findings also provide intriguing implications for neurorehabilitation aimed at long-lasting performance improvements that generalize beyond a clinical setting. They suggest that therapists should consider incorporating tasks or situations that elicit a threatening physical consequence if the patient moves incorrectly or in manner inconsistent with how a therapist is training them. Safety is paramount in these cases, which can be managed using fall safety harness systems and/or virtual reality.

## Synthesis

Reviewing Editor: Satu Palva, Helsingin yliopisto Decisions are customarily a result of the Reviewing Editor and the peer reviewers coming together and discussing their recommendations until a consensus is reached. When revisions are invited, a fact-based synthesis statement explaining their decision and outlining what is needed to prepare a revision will be listed below. The following reviewer(s) agreed to reveal their identity: NONE.

Synthesis

The present manuscript describes a well-designed study exploring how increasing the consequences of movement errors affects motor learning memory (inter-limb transfer and generalizability to another task). Healthy young adult participants adapted to wearing prism glasses whilst performing a precision stepping task, whereby the movement error (large lateral misplaced step) could be accompanied by a threat to balance (slip). The novel findings revealed that the group who performed the task with the consequence of a slip demonstrated both increased interlimb-transfer and generalization to a different (obstacle stepping) gait task. This group also demonstrated increased savings when performing the adapted task 1-week later.

They make a compelling argument with regard to the motivation of the study, as it seems intuitive that actions with more salient consequences-particularly ones with a steep cost-should be remembered better. The data are very convincing, as it is clear that the group experiencing slips during adaptation demonstrated behavior consistent with increased generalization and savings.

While the motivation, much of the study design, and results are clear, the interpretation of the results and their placement, by the authors, within the broader motor learning literature should be enhanced.

. On the other hand, if the authors believe that the slip perturbation could not simply be replaced by another salient cue or form of feedback, they should attempt to explain their position more thoroughly throughout the text.

This is an eloquently designed study - and one that makes considerable contributions to the research area as a whole. The findings are reported clearly and the paper is well-written throughout.

Major

Are the effects observed during Consequence condition driven by the physical consequences associated with the error (i.e., the risk of a slip); or whether the physical consequences (the slip itself) drive the enhanced motor learning observed through increasing the error signal itself?

The findings should be explained in terms of prior literature on recall of explicit strategies and explicit learning generalizes more broadly than implicit learning (Morehead et al 2015, Haith et al 2015, Poh et al 2021).

Providing a schematic or flow chart illustrating the conceptual framework would be very helpful.

There are clear predictions presented in Figure 1 (points of divergence between blue and orange functions and dots), but little discussion of the theoretical or empirical basis for these very specific predictions. For example, why should there be improved generalization and savings for the consequence group, but no difference in interference on the opposite perturbation?

- The rationales for the generalization tasks seemed ad hoc. This made it hard to assess what the authors’ predictions were for the consequence group as compared to controls, and why? Could the authors bolster their rationale and explanation for why a physical consequence should translate into better (or worse) generalization on these specific tasks? What conceptual framework are the authors relying on?

- Although the authors state that there were no differences between groups across session 1’s testing phases (L366), the learning functions look qualitatively different (consequence group’s learning function has a sharper elbow), and in figure 5, one can see that the adaptation rate appears faster for the consequence group. As these results could be viewed through the lens of formation of an explicit strategy, they seem worthy of further elaboration/discussion.

- What do the data on each trial leading up to the target step look like? That is, are there differences in how the two groups approach the target in the steps leading up to the target? Those data seem important to report, at least in summary format, and may also impact the overall interpretation of the results..

- L92-95: What does “mixed evidence of support for both generalization...” mean? Are you saying the combined results of these studies call into question the presence of generalization and consolidation? That is what mixed evidence sounds like to me; however, one could easily argue that the literature provides many examples of robust generalization of adaptation and consolidation.

Minor

- Line 116 - Did all participants adhere to stepping into the target with the correct foot?

- You state that participants were instructed to step onto the projected target “without stopping” (line 117). From experience using precision stepping paradigms, participants typically will increase their stance time in the step prior to the precision step. How did you confirm that participants did indeed adhere to this instruction?

- Line 146 - You may well describe this later, but did participants complete the generalization testing whilst also wearing the prism glasses?

- Line 175 - Was this exactly 1-week for all participants?

- Line 199 - Can you please clarify what is meant by “late prism adaptation”?

- Given that the stepping target was projected onto the floor rather than being for example a raised target (e.g., the classic Mark Hollands paradigm), would a misplaced step whilst wearing the prism glasses would carry a detectable stepping error signal?

- Fig. 3 - Is this ML error? Fig. 2 before listed this as the direction of the error.

- It is a shame that there were no state physiological (e.g., arousal) or psychological measures collected (e.g., anxiety, fear of falling etc.) as these could have shed further light on the potential processes driven these between-group effects.

- Line 544-552. The prior studies on is well accepted that postural threats increase the reliance on conscious processes to prevent errors when performing both quiet standing balance should be cited. (https://www.nature.com/articles/s41598-020-79417-5, https://pubmed.ncbi.nlm.nih.gov/19729308/ , https://www.sciencedirect.com/science/article/pii/S0966636215010012) and precision stepping gait tasks (https://psycnet.apa.org/fulltext/2019-28024-001.html , https://academic.oup.com/psychsocgerontology/article/75/9/1911/5881747).

- Is the target available throughout the duration of the trial?

- L180-183: It took me several reads to understand the block structure of the task (despite the figure). I think it was because baseline phases more typically immediately precede an associated perturbation block, but here three different baseline phases are performed immediately preceding the prism perturbation block. I think the writing could be clearer here and throughout the description of the experimental paradigm.

- As an exploratory analysis, it would be interesting to provide a visualization of the number/intensity of the slips per subject against some of the primary metrics for generalization and savings. I think a simple scatterplot would be a nice addition and satisfy many readers’ curiosity about this.
